# Supplementary figures and images for: Comparisons between dipeptidyl peptidase-4 inhibitors and other classes of hypoglycemic drugs using two distinct biomarkers of pancreatic beta-cell function: A meta-analysis
Source: PLoS One. 2020 Jul 24;15(7):e0236603. doi: 10.1371/journal.pone.0236603 (PMC7380634; doi:10.1371/journal.pone.0236603)

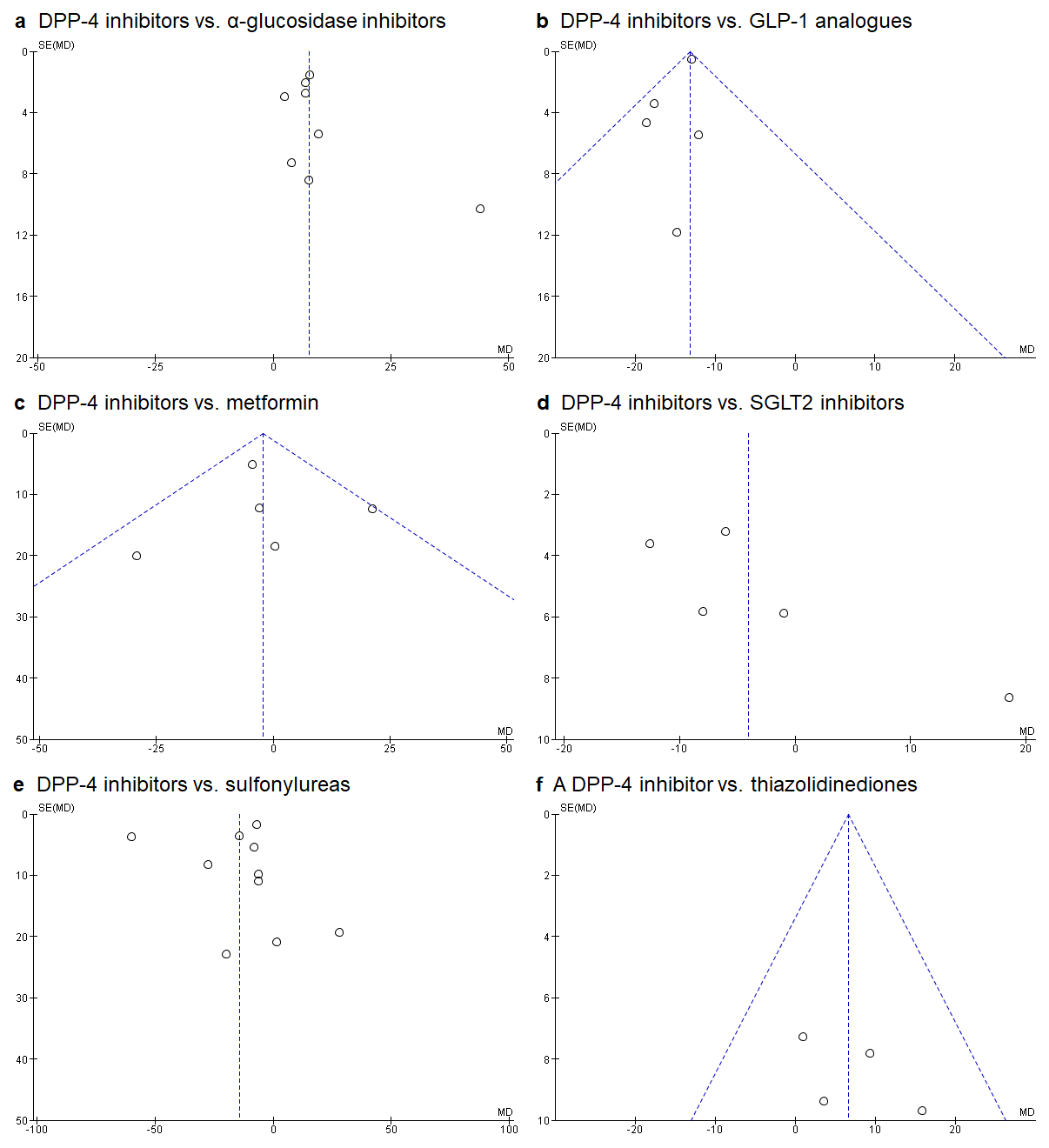

Supplement: S1 Fig — This plot is effect size (weighted mean difference of homeostatic model of assessment for beta-cell function [HOMA-β] from individual articles) (X axis) versus standard error of effect size (Y axis). Dotted vertical line represents pooled estimate of effects. Abbreviations: DPP-4, dipeptidyl peptidase-4; GLP-1, glucagon-like peptide-1; MD, mean difference; SE, standard error; SGLT2, sodium-glucose cotransporter 2. (TIF) [file pone.0236603.s001.tif]

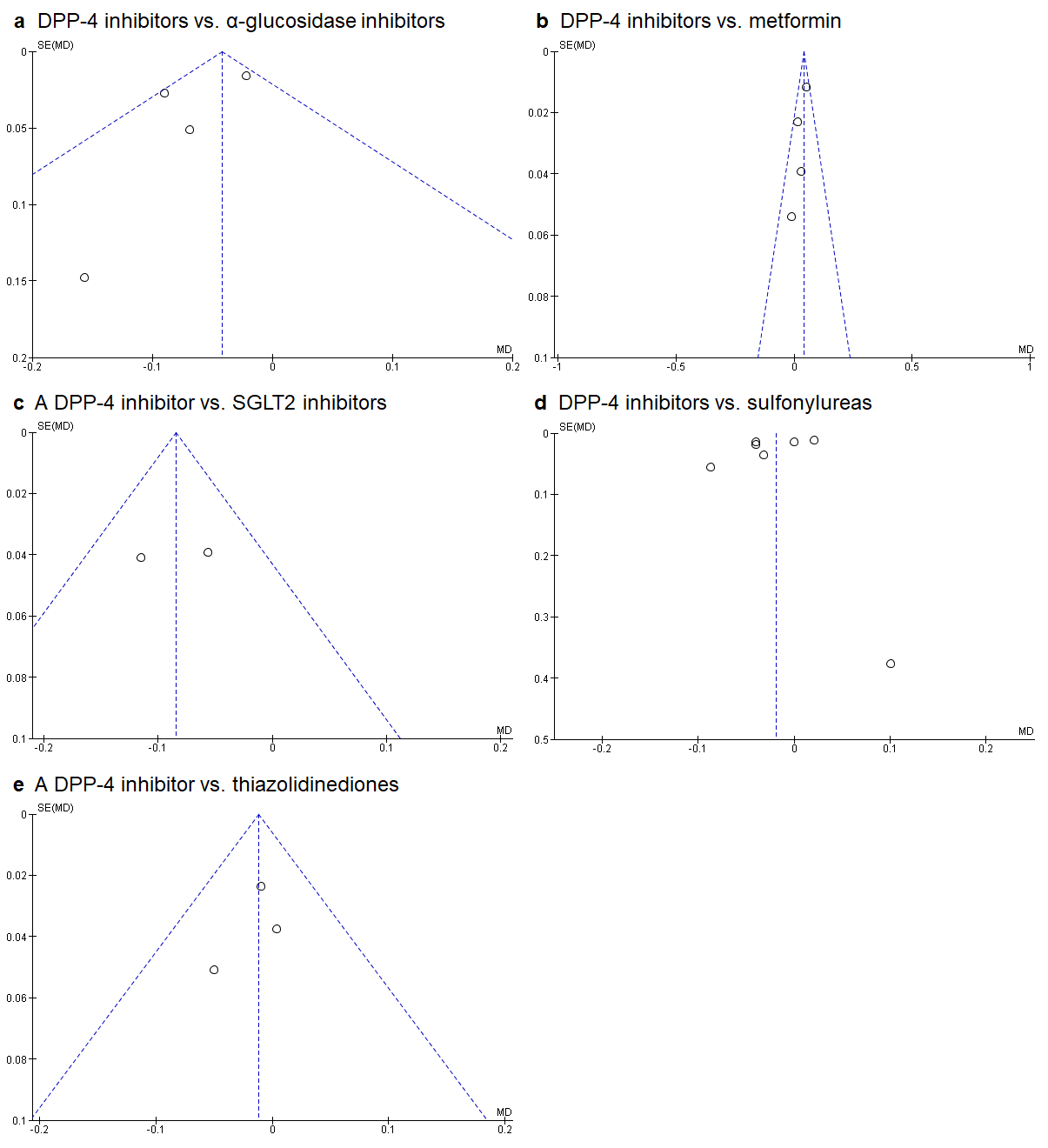

Supplement: S2 Fig — This plot is effect size (weighted mean difference of proinsulin-to-insulin ratio [PIR] from individual articles) (X axis) versus standard error of effect size (Y axis). Dotted vertical line represents pooled estimate of effects. Abbreviations: DPP-4, dipeptidyl peptidase-4; MD, mean difference; SE, standard error; SGLT2, sodium-glucose cotransporter 2. (TIF) [file pone.0236603.s002.tif]
