# Supplementary material for: Comparisons between dipeptidyl peptidase-4 inhibitors and other classes of hypoglycemic drugs using two distinct biomarkers of pancreatic beta-cell function: A meta-analysis
Source: PLoS One. 2020 Jul 24;15(7):e0236603. doi: 10.1371/journal.pone.0236603 (PMC7380634; doi:10.1371/journal.pone.0236603)
Supplement: S2 Table — (DOCX) [file pone.0236603.s004.docx]

1. MEDLINE search strategy

| **DPP-4 inhibitors** | **Disease name** | **Publication**  **type** | **Drug name** | **Search formula** |
| --- | --- | --- | --- | --- |
| alogliptin | #1  "diabetes mellitus"[MeSH Terms] OR  ("diabetes"[All Fields] AND  "mellitus"[All Fields]) OR  "diabetes mellitus"[All Fields] | #2  Randomized  Controlled  Trial[ptyp] | #3  "alogliptin"[Supplementary Concept] OR  "alogliptin"[All Fields] | #1 AND #2 AND #3 |
| anagliptin |  |  | #4  "anagliptin"[Supplementary Concept] OR  "anagliptin"[All Fields] | #1 AND #2 AND #4 |
| linagliptin |  |  | #5  "linagliptin"[MeSH Terms] OR  "linagliptin"[All Fields] | #1 AND #2 AND #5 |
| saxagliptin |  |  | #6  "saxagliptin"[Supplementary Concept] OR  "saxagliptin"[All Fields] | #1 AND #2 AND #6 |
| sitagliptin |  |  | #7  "sitagliptin phosphate"[MeSH Terms] OR  ("sitagliptin"[All Fields] AND  "phosphate"[All Fields]) OR  "sitagliptin phosphate"[All Fields] OR  "sitagliptin"[All Fields] | #1 AND #2 AND #7 |
| teneligliptin |  |  | #8  "3-(4-(4-(3-methyl-1-phenyl-1H-pyrazol-5-yl)piperazin-1-yl)pyrrolidin-2-ylcarbonyl)thiazolidine"[Supplementary Concept] OR  "3-(4-(4-(3-methyl-1-phenyl-1H-pyrazol-5-yl)piperazin-1-yl)pyrrolidin-2-ylcarbonyl)thiazolidine"[All Fields] OR  "teneligliptin"[All Fields] | #1 AND #2 AND #8 |
| vildagliptin |  |  | #9  "vildagliptin"[MeSH Terms] OR  "vildagliptin"[All Fields] | #1 AND #2 AND #9 |

2. CENTRAL search strategy

| **DPP-4 inhibitors** | **Disease name** | **Publication**  **type** | **Drug name** | **Search formula** |
| --- | --- | --- | --- | --- |
| alogliptin | #1  "diabetes mellitus"[All Text]  (Word variations have been searched) | #2  "randomized controlled trial":pt  (Word variations have been searched) | #3  "alogliptin"[All Text]  (Word variations have been searched) | #1 AND #2 AND #3 |
| anagliptin |  |  | #4  "anagliptin"[All Text]  (Word variations have been searched) | #1 AND #2 AND #4 |
| linagliptin |  |  | #5  "linagliptin"[All Text]  (Word variations have been searched) | #1 AND #2 AND #5 |
| saxagliptin |  |  | #6  "saxagliptin"[All Text]  (Word variations have been searched) | #1 AND #2 AND #6 |
| sitagliptin |  |  | #7  "sitagliptin"[All Text]  (Word variations have been searched) | #1 AND #2 AND #7 |
| teneligliptin |  |  | #8  "teneligliptin"[All Text]  (Word variations have been searched) | #1 AND #2 AND #8 |
| vildagliptin |  |  | #9  "vildagliptin"[All Text]  (Word variations have been searched) | #1 AND #2 AND #9 |

3. Ichushi-web search strategy

| **DPP-4 inhibitors** | **Disease name** | **Publication type** | **Drug name** | **Search formula** |
| --- | --- | --- | --- | --- |
| alogliptin | #1  "diabetes mellitus" | #2  “randomized controlled trial” | #3  "alogliptin" | #1 AND #2 AND #3 |
| anagliptin |  |  | #4  "anagliptin" | #1 AND #2 AND #4 |
| linagliptin |  |  | #5  "linagliptin" | #1 AND #2 AND #5 |
| saxagliptin |  |  | #6  "saxagliptin" | #1 AND #2 AND #6 |
| sitagliptin |  |  | #7  "sitagliptin" | #1 AND #2 AND #7 |
| teneligliptin |  |  | #8  "teneligliptin" | #1 AND #2 AND #8 |
| vildagliptin |  |  | #9  "vildagliptin" | #1 AND #2 AND #9 |
