# Supplementary material for: Comparisons between dipeptidyl peptidase-4 inhibitors and other classes of hypoglycemic drugs using two distinct biomarkers of pancreatic beta-cell function: A meta-analysis
Source: PLoS One. 2020 Jul 24;15(7):e0236603. doi: 10.1371/journal.pone.0236603 (PMC7380634; doi:10.1371/journal.pone.0236603)
Supplement: S3 Table — Symbols: +, low risk of bias; -, high risk of bias; ?, unclear risk of bias. (DOCX) [file pone.0236603.s005.docx]

| **Study** | **Random sequence**  **generation** | **Allocation concealment** | **Blinding of participants**  **and personnel** | **Blinding of outcome**  **assessment** | **Incomplete outcome data** | **Selective reporting** | **Other bias** |
| --- | --- | --- | --- | --- | --- | --- | --- |
| Pratley 2014 [29] | ? | ? | + | + | + | + | + |
| Seino 2011 [30] | + | ? | + | ? | + | ? | ? |
| Kaku 2012 [31] | ? | ? | ? | ? | + | ? | ? |
| Forst 2010 [32] | ? | ? | + | ? | - | + | + |
| Gallwitz 2012 [33] | + | + | ? | + | + | + | + |
| Kawamori 2012 [34] | ? | ? | ? | + | + | + | ? |
| Du 2017 [35] | + | + | - | - | + | + | + |
| Ekholm 2017 [36] | ? | ? | + | + | ? | + | ? |
| Göke 2010 [37] | + | + | ? | ? | + | ? | + |
| Tao 2018 [38] | + | ? | - | - | + | + | + |
| Aschner 2010 [39] | + | ? | + | + | - | + | ? |
| Berg 2011 [47] | ? | ? | ? | ? | - | ? | ? |
| Derosa 2010 [40] | + | ? | ? | ? | + | ? | ? |
| Derosa 2013 [48] | + | ? | + | + | + | ? | ? |
| Arjona 2013a [45] | + | ? | + | ? | + | + | ? |
| Arjona 2013b [46] | + | ? | + | + | - | + | + |
| Fukui 2015 [57] | ? | ? | - | - | + | ? | ? |
| Gadde 2017 [58] | ? | + | - | + | + | + | ? |
| Henry 2014 [49] | ? | ? | + | ? | ? | + | ? |
| Iwamoto 2010 [41] | + | ? | ? | ? | - | ? | ? |
| Kobayashi 2014 [50] | + | ? | - | - | + | + | - |
| Park 2017 [59] | ? | ? | - | - | ? | + | ? |
| Pérez-Monteverde 2011 [51] | ? | ? | + | ? | ? | + | ? |
| Pratley 2011 [52] | ? | ? | - | - | + | + | ? |
| Pratley 2018 [60] | + | + | + | + | + | + | + |
| Rosenstock 2012 [53] | ? | ? | + | + | + | + | ? |
| Schernthaner 2013 [54] | + | + | + | ? | + | + | + |
| Scott 2007 [43] | + | ? | ? | + | + | ? | ? |
| Scott 2008 [42] | ? | ? | + | + | + | + | ? |
| Seck 2010 [44] | + | ? | + | ? | - | + | ? |
| Shi 2019 [64] | + | + | - | + | ? | ? | ? |
| Takihata 2013 [55] | + | + | - | - | + | + | ? |
| Tsurutani 2018 [61] | + | ? | - | - | + | + | ? |
| Weinstock 2015 [62] | + | ? | + | + | + | - | ? |
| Williams-Herman 2010 [56] | + | ? | + | + | + | + | ? |
| Yokoh 2015 [63] | + | + | - | - | + | + | - |
| Iwamoto 2010 [65] | ? | ? | ? | ? | + | ? | + |
| Kim 2017 [66] | ? | ? | - | - | + | + | ? |
| Sawayama 2013 [67] | + | ? | ? | ? | + | ? | ? |
| Takeshita 2015 [68] | + | ? | - | - | + | ? | ? |

Symbols: +, low risk of bias; -, high risk of bias; ?, unclear risk of bias
